# Anæsthetics

**Published:** 1905-03-04

**Authors:** 


### Progress in Medicine and Surgery.

### ANÆSTHETICS.

(*Continued from page* 389.)

**Delayed Chloroform Poisoning.**—This condition as met with in children and originally described by Guthrie 10 years ago has been further investigated by Stiles and McDonald.[16] Two cases are described by these authors in which death occurred three days and 26 hours respectively after the administration of chloroform. Fat embolism, carbolic acid poisoning and sepsis were excluded. Though but few cases have been recorded in this country many have been noted in Germany, but unfortunately for the most part sepsis could not be excluded. The anatomical changes found are fatty degeneration of the liver, with degenerative changes in the renal tubules and evidences of a general toxic condition. The symptoms are persistent vomiting, often hæmorrhagic, a small, rapid, often irregular pulse, and extreme restlessness and collapse. The authors consider that experimental evidence points to these changes being the direct result of the chloroform poisoning on previously healthy tissues, the main predisposing cause being idiosyncracy. They recommend rectal and intravenous injections, lavage of the stomach followed by small doses of mercury and cautious stimulation. They agree with Guthrie in discountenancing morphia. Watson Cheyne[17] sees great difficulty in attributing these results to the chloroform owing to the small dosage of the drug given in some cases, and Guthrie[18] in view of this fact considers that they are due to the action of the anæsthetic on previously diseased tissues. He considers that where there are symptoms pointing to deranged action of the liver ether should be employed. McCardie[19] quotes two cases of Forster's in which similar changes were observed, and suggested that lymphatism might be a contributing factor, and Burghard[20] notes that in one of Guthrie's cases the ill effects were only observed after a second administration of the anæsthetic. Brackett, Stone, and Low[21] have recorded a series of cases in which death occurred in children suffering from severe vomiting and collapse in which the odour of acetone was noticed in the breath, and the presence of that body determined in the urine. Most of these occurred after the administration of chloroform or ether; in one fatal case and two which recovered, however, no operation had been performed. Fatty degeneration of the liver, kidneys, and muscles was found in the fatal cases. Becker[22] is quoted as having observed three cases of diabetes in which operation was followed by severe and fatal symptoms, and frequently found acetonuria in healthy persons after anæsthesia. Bicarbonate of soda with stimulants is said to have a good effect in such cases.

**Massage of the Heart.**—A case is reported by Keen[23] in which the patient, who was known to take chloroform badly, was operated on for total extirpation of the larynx. Chloroform and oxygen were given, and though the anæsthesia was as light as possible, he was cyanosed throughout, and his pulse suddenly failed as the wound was being closed. Artificial respiration for ten minutes with the administration of oxygen and strychnine failed to improve the condition, so the abdomen was opened and for nearly half-an-hour the heart was kneaded between the two hands, one on the chest and one below the diaphragm. The patient did not recover, but in a case quoted of Ingelsrud's two minutes' massage of the heart itself (the pericardium being opened) restored the rhythmical action of the organ and recovery ensued.

**Ethyl Chloride.**—A simple apparatus for administering this anæsthetic has been described by Kitchin.[24] An ordinary Clover's inhaler is used, the ebony plug which stops the opening by which the ether chamber is filled, being replaced by a cork, through which a hole $\frac{1}{4}$ inch in diameter has been bored. The index is turned to mark 2, and after a few respirations into the bag, the necessary amount of ethyl chloride is sprayed through the hole in the cork, which is then closed by the finger. The anæsthetic is thus gradually given, which prevents coughing and struggling, and the metal ether chamber rapidly evaporates it and obviates freezing. The ethyl chloride, moreover, cannot run down on to the face-piece and spoil the rubber. Ether can easily be made to follow if longer anæsthesia is required. Daniell[25] finds that the metal ether chamber imparts an unpleasant taste to the vapour of ethyl chloride, and advises a modification of Hewitt's apparatus. This consists of a graduated glass tube into which the ethyl chloride is measured, and which is attached by a tap to the angle-mount of a Clover's inhaler intended for the gas-ether sequence. The tap is turned off and the tube containing the ethyl chloride allowed to hang down. After adjustment of the mask, the tap is turned on and the tube gradually rotated so that the anæsthetic runs into the rubber bag. By this means a weak vapour can at first be given, no freezing near the patient's face can take place, and exact dosage is secured. Gas may be given first for a few seconds and then the ethyl chloride tube be substituted; or the ether chamber may be placed in position charged so as to give an ethyl-ether sequence. In this case only 1 5-2cm. ethyl chloride are required, and ether should be turned on quite early (after a few seconds) to allow of an admixture of the vapours. Daniell has used this apparatus 500 times. Porritt[26] interposes a tube between the bag and face-piece or the bag and ether chamber of a Clover's inhaler inside which is a piece of lint on to which ethyl chloride is sprayed through a small hole, closed at will by a revolving collar. He claims for this arrangement much the same advantages as are given by Daniell's method, and like him insists on the importance of giving ether early in a mixed anæsthesia, so that both vapours are inhaled at once. Owing to the fact that the hole in the tube can be kept open, the apparatus can be used for chloroform or A.C.E mixture, but Porritt finds that a conscious interval always occurs with this sequence, and that vomiting and after-effects are always more severe than with ethyl-ether.

**Hyoscine Hydrobromide.**—Both morphia and atropine have been used hypodermically before ether anæsthesia to ensure quiet induction, to prevent salivation, and to minimise the quantity of ether used. Roberston[27] points out the disadvantages of both these drugs, as being likely to increase nausea and vomiting, and to depress the nervous centres to a dangerous degree. He has in 57 cases successfully administered $\frac{1}{100}$ gr. hyoscine hydrobromide. The drug must be pure and prepared from hyoscyamus niger. It is given hypodermically half an hour before ether is to be administered. Thirst, dryness of the mouth, and drowsiness usually occur soon after the injection; the induction of anæsthesia is rapid and quiet; in the great majority of cases there is no salivation and no vomiting or nausea afterwards. Very little ether is necessary to maintain anæsthesia and no dangerous or unpleasant symptoms referable to the hyoscine were noticed in any case. Robertson gives an account of the physical and physiological properties of the drug, which are very similar to those of atropine, except that it lowers the irritability of the higher cerebral centres. He has also collected a number of cases of poisoning by the drug. Only two of these were fatal, and in one of them it is doubtful whether death was due to the drug or to perforation of a typhoid ulcer. In most of the other cases large doses had been given, or the drug had been combined with other narcotics or sedatives to which a part of the dangerous symptoms could be ascribed. He concludes that ordinary doses are not dangerous but admits that a few individuals show a marked susceptibility similar to that exhibited by some towards atropine.

[16] Scot. Med. and Surg. Jour., Aug. 1904, p. 97. [17] Lancet, June 11, 1904, p. 1658. [18] Ibid. [19] Ibid. [20] Ibid. [21] Ibid., Sept. 17, 1904, p. 846. [22] Ibid. [23] Med. Rec., July 2, 1904, p. 23. [24] Brit. Med. Jour., Dec. 26, 1903, p. 1639. [25] Ibid., April 23, 1904, p. 949. [26] Ibid., July 9, 1904, p. 66. [27] Med. Rec., Jan. 9, 1904, p. 52.

